# Patients' Length of Stay in Women Hospital and Its Associated Clinical and Non-Clinical Factors, Tehran, Iran

**Published:** 2011-05-01

**Authors:** R Ravangard, M Arab, H Zeraati, A Rashidian, A Akbarisari, F Mostaan

**Affiliations:** 1 Department of Health Management and Economics, Tehran University of Medical Sciences, Tehran, Iran; 2 Department of Epidemiology and Biostatistics, School of Public Health, Tehran University of Medical Sciences, Tehran, Iran; 3 Department of Obstetrics and Gynecology, School of Medicine, Imam Khomeini Hospital, Tehran University of Medical Sciences, Tehran, Iran

**Keywords:** Length of stay, Women hospital, Clinical and non-clinical factors

## Abstract

**Background:**

Length of Stay (LOS) is an appropriate hospital indicator to evaluate hospital resource utilization rate, efficiency, and quality of services delivered. In this survey, we aimed to study hospital LOS and determine its association with clinical and non-clinical factors in Women Hospital in Tehran.

**Methods:**

In this cross-sectional study, we reviewed all 3421 charts of patients admitted in Oncology, Surgery and Obstetrics units in 2008. We used a data collection sheet and conducted interviews to collect the following data: distance from living area, medical insurance coverage types, admission and discharge months, days and times, inpatient units, final diagnoses and the number of diagnostic tests.

**Results:**

The overall median of the LOS in the studied hospital was 50.8 hours. The medians were 48.5, 54.4, and 94.2 hours in the Obstetrics, Surgical and Oncology units, respectively. Results showed that the associated factors with the LOS were patient admissions on Thursdays, admitting by residents, the number of performed diagnostic tests (p<0.001), suffering from neoplastic diseases (p=0.005) and spouse jobs.

**Conclusion:**

Among the associated factors, policy makers and managers can only change the admission days and the number of diagnostic tests to decrease the LOS. Further researches are needed to find other factors associated with LOS.

## Introduction

In healthcare, a productive management is the one by which healthcare services are planned and controlled cost-effectively and all organizational goals are achieved wherein the quality is maintained. Achieving the goals is possible through utilizing resources properly, controlling hospital admissions and inpatient Length of Stay (LOS), as well as using ancillary services and equipments properly.

LOS is a hospital indicator usually reported as percent. LOS indicates resource utilization in hospitals and presents inpatient days by which efficiency and quality of services delivered are evaluated. LOS varies among various hospital units; for example, LOS is relatively short in Pediatrics Unit, while it is much longer in Geriatrics' wards.[1] The LOS includes the time between patient's admission and discharge time and measures both bed utilization and inpatient units' efficiency.[2]

The LOS has been decreased considerably over the past years both overall and for most diseases. In many countries, healthcare providers have been under much political and managerial pressure to keep LOS in a desirable minimum level. Some studies have shown that LOS reduction would reduce costs without compromising patients' outcomes.[3] In other words, hospital managers, policy makers and third party payers continue to be interested in shortening hospital stays as a main strategy for hospital cost-containment and efficient utilization of scarce hospital resources. However, it should be noticed that LOS reduction level is restricted by factors such as quality and effectiveness considerations. It means that the average LOS should be decreased to a level in which service quality and patients' outcomes are not compromised. To achieve this goal, it is important to know more about the factors that play a significant role in decreasing patients' LOS.[4] There is no general agreement on the list of effective factors which shorten the LOS. However, four following categories of the LOS determinants have been identified: 1) Patient-related factors including age, sex, social support status, socio-economic status, nutritional status, hospital acquired infections and post operative infections; 2) Hospital-related factors including hospital size, nurse-patient ratios and area of residence; 3) Sources and types of payment including insurance coverage, types of hospital payments; 4) Physician-related factors including the kind of physicians' practice as a general practitioner, private physician or house staff physician.[5] In another categorization, these factors have been divided into two groups: 1) Supply factors which depend on circumstances related to the provider of care-for instance, bed availability and supply, payment methods, and discharge policies; 2) Demand factors which depend on issues related to patients' needs; for instance, disease severity, co-morbidity, and direct or indirect costs to the patients or their careers.[3]

It is worthy noticing that both over-hospitalization and under-hospitalization have adverse impacts on costs and quality of care provided. Over-hospitalization is not only expensive because of using expensive and scarce hospital resources, but also can be harmful to health because of carrying the risks of nosocomial infections, and iatrogenic complications. On the other hand, under-hospitalization, whether related to inappropriate clinical management or due to cost-containment, may have effects on quality of care provided and results in unsatisfactory outcomes.[5]

Previous studies have shown that factors such as age,[6][7][8][9][10][11][12][13][14][15][16][17][18][19] sex,[5][6][10] marital status,[9] race/ethnicity,[11][13] patients' origin,[9][20] residency location,[9][10][21] socio-economic status,[19] insurance status, source and types of payment,[5][11][13][17] hospital types,[4][5][17][19][22] hospital size,[4][5][17][23] the month, day and time of patient admission,[4][9][15] admission types in terms of being elective, urgent or emergency,[7] patients' physical and functional status,[12][13][15][18] hospitalization experience,[7] discharge destination,[4][10][13] patients' status at discharge time,[7] hospitalizing physician's academic degree[24] and types and severity of illnesses[8][13][21][25][26] have impacts on inpatient length of stay.

Studies conducted in Iran to review hospital LOS either have been carried out on insufficient samples; therefore they do not have reliable results such as Faraji Khiavi's[8] and Gholi vahidi's[7] studies or have aimed to compare different statistical methods used for modeling inpatient LOS such as Salesi's[21] and Rafie's[9][10] studies. As there are no comprehensive studies on the LOS in Iran to assess the related factors and there is increasing need for implementing effective management and proper utilization of scarce hospital resources, especially beds which are the most important hospital resources, this survey aimed to study the patients' hospital LOS and its related clinical and non-clinical (demographic and hospital-related) factors in Women Hospital (a tertiary teaching hospital) in Tehran in order to help managers to plan the use of available resources properly.

## Materials and Methods

In this cross-sectional survey, we reviewed all 3421 charts of patients admitted and hospitalized in Women Hospital in Tehran in 2008. This hospital is a tertiary specialty teaching hospital which has several units including Obstetrics, Surgery, and Oncology, High risk infants, NICU, Phototherapy and Obstetrics Emergency. In this study, we only studied women's related units which were Obstetrics, Surgery and Oncology units. These units had 20, 33 and 12 available beds, respectively. In 2008, 1704 (49.8%), 1360 (39.8%) and 357 (10.4%) charts belonged to the Obstetrics, Surgery and Oncology units, respectively.

The required data (such as marital status, age, distance from living area, medical insurance coverage types, admission and discharge months, days and times, inpatient units, related physician specialties and academic degrees, final diagnoses, the number of diagnostic tests such as laboratory tests, radiographies and sonographies, admission types, patients' status at discharge time, the number of previous hospitalization and hospital costs) were collected using a data collection sheet. In order to collect other data which were not recorded in the patients' charts (such as patients and their spouse jobs, educational levels and the family income), we conducted interviews with all inpatients for three months (winter months) which exceeded 951 interviews and the related analyses were done only for these patients. In order to categorizing final diagnoses, we used ICD-10.

Kolmogrov-Smirnov test showed that the distribution of the LOS data was not normal. Therefore, the relationships between the LOS and related factors were studied using nonparametric tests such as Mann-Whitney, Kruskal-Walis and Spearman correlation tests. Finally, because the residuals of LOS data were normal, we used Multiple Linear Regression to study the factors that affect LOS simultaneously. P<0.05 was considered statistically significant. The study protocol and ethical aspects were approved by the Ethics Committee of Tehran University of Medical Sciences.

## Results

The overall median of the LOS was 50.8 hours in the hospital. The medians for the Obstetrics, Surgical and Oncology units were 48.5, 54.4 and 94.2 hours, respectively. We analyzed all factors without any previous assumptions about which ones had association with LOS. As we see in Table 1 and Table 2, there was a significant relationship between the LOS with the marital status (the unmarrieds' LOS was longer than the married ones), medical insurance coverage types, type of related physicians' specialties, related physicians' academic degrees (patients who had been admitted by residents had shorter LOS), admission types (elective patients had longer LOS), patients' status at discharge, final diagnoses (p<0.001) and the spouse jobs (p=0.009).

**Table 1:** The distribution of patients’ length of stay (hour) based on non-clinical (demographic and hospital related) factors in Women Hospital in Tehran

**Factors**	**Median**	**Percentile 5**	**Percentile 95**	**Test Results**	**Factors**	**Median**	**Percentile 5**	**Percentile 95**	**Test Results**
Marital Status					Admission Days				
Married	50.6	23.5	197.4	Z^a^= 7.080 *P* value< 0.001	Saturday	51	23.6	189.4	X^2^= 24.402 *P* value< 0.001
Unmarried	76.8	25.1	296.3		Sunday	50.7	23.6	192.6	
Medical Insurance Coverage Types					Monday	50.2	23.4	263.1	
Insurance on the Bed	50	23.5	163.4	X^2^^b^ = 53.088 *P* value< 0.001	Tuesday	49.5	22.8	179.4	
Social security Insurance	51	23.6	220.8		Wednesday	54.7	22.2	244.4	
Uninsured Patients	48.7	21.9	145		Thursday	53.8	24.1	243.1	
Medical Services Insurance	52	24.1	240.2		Friday	50.2	24.3	146.3	
Rural Insurance	72.6	22.5	288.4		Patients' status atdischarge				
Forces Insurance	69.8	24.6	231.2		Recovery status	50	22.7	15.2	X^2^= 79.203 *P* value< 0.001
Other types of Insurance	50.6	22.6	269.1		Relative recovery status	53.3	24.5	266.5	
Spouse Jobs					Leaving Against Medical Advice (LAMA)	48.5	21.6	193	
Worker, Farmer, Stockbreeder or Retired	61.7	24.7	439.8	X^2^= 13.443 *P* value= 0.009	Need to Follow up after Discharge from Hospital	52	23.8	259	
Private and Public Employee and Military	52.9	22.1	242.1		Related Physicians' Academic Degrees				
Healthcare Jobs	45.1	23.6	74.5		Residents	50.2	22.7	196	Z= 8.045 *P* value< 0.001
Educational Jobs	52.4	22.6	336.7		Attending Physicians	52.2	25.6	240.8	
Other Jobs	51.1	22.8	222.2		Admission Type				
Inpatient Units					Elective	51.8	24.6	238.6	Z= 9.960 *P* value< 0.001
Obstetrics	48.5	22.2	13.6	X^2^= 357.225 *P* value< 0.001	Emergency	49.1	22.4	185.6	
Surgery	54.4	24.8	238.8						
Oncology	94.2	25.9	334.3						
Related Physician Specialty									
Gynecologist	50.5	23.4	194.5	X^2^= 163.571 *P* value< 0.001					
Internist	145.3	46.9	333						
General Surgeons	51.7	27.4	140.2						

^a^ Mann-Whitney Test

^b^ Kruskal-Wallis Test

**Table 2:** The distribution of the patients’ length of stay (hour) based on the final diagnoses (clinical) categories in Women Hospital in Tehran

**Factors**		**Median**	**Percentile 5**	**Percentile 95**	**Test Results**
Final Diagnoses Categories					
	1) Pregnancy, Childbirth and Peurperium Diseases	49.1	22.5	150.8	X^2^= 291.459 P-value< 0.001
	2) Neoplasms	79.3	25	329.1	
	3) Endocrine, Nutritional and Metabolic Diseases	77	25.9	350.4	
	4) Digestive System diseasesِ	120.1	49.3	335	
	5) Genitourinary System Diseases	51.4	25.2	220.5	
	6) Other Diseases	51.8	24.1	231.2	

This study showed that the difference between the LOS and spouse jobs was related to longer LOS in patients whose spouses were workers, farmers, stockbreeder or were retired (med=61.5 h) compared with those who had private and public jobs, military employments (med=52.9 h) or the other jobs (med=51.1 h) (p<0.005).

There was a difference between the LOS and the types of insurance coverage. The LOS was shorter in the patients who had insurance on the bed type (med=50 h) compared with those who had the social security insurance (med=51 h), medical services insurance (med=52 h) or rural insurance (med=72.6 h). Patients who had no insurance coverage had also shorter LOS (med=48.7 h) compared with those who had rural insurance (med=72.6 h), social security insurance (med=51 h), medical services insurance (med=52 h), armed forces insurance (med=69.8 h) or the other types of insurance (med=50.6 h). Also, patients who had social security insurance (med=51 h) had shorter LOS compared with those who had rural insurance (med=72.6 h) (p<0.05).

The LOS was longer in the Oncology Unit (med=94.2 h) compared with that in the Surgery (med=54.4 h) and Obstetrics (med=48.5 h) units. The LOS was also longer in the Surgery Unit (med=54.4 h) compared with that in the Obstetrics' one (med=48.5 h) (p<0.001). The LOS was shorter in the patients who were admitted on Fridays (med=50.2 h), which are weekends, compared with those who were admitted on Wednesdays (med=54.7 h) and Thursdays (med=53.8 h). Moreover, the LOS was longer in the patients who were admitted on Wednesdays (med=54.7 h) compared with those who were admitted on Saturdays (med=51 h) and Tuesdays (med=49.5 h) (p<0.05). Since there was a steady influential trend on the LOS in admission days, patients who were admitted on Wednesdays had longer LOS compared with those who were admitted on the other days of the week.

The LOS was more than twice longer in patients who were admitted by internists (med=145.3 h) compared with those who were admitted by the Gynecologists (med=50.5 h) and general surgeons (med=51.7 h) (p<0.001). The LOS was shorter in patients who were discharged with recovery status (med=50 h) compared with those who were discharged with relative recovery status (med=53.3 h) and those need to be followed up after discharge from the hospital (med=52 h). The LOS was shorter in patients who were left against medical advice (LAMA) (med=48.5 h) compared with those discharged with relative recovery (med=53.3 h) and those need to be followed up after discharge from the hospital (med=52 h) (p<0.001).

Finally, the LOS was shorter in patients who had pregnancy, childbirth and peurperium diseases (med=49.1 h) compared with those who had neoplasm (med=79.3 h), endocrine, nutritional and metabolic diseases (med=77 h), digestive system diseases (med=120.1 h), genitourinary system diseases (med=51.4 h) and other diseases (med=51.8 h). The LOS was shorter in patients who had genitourinary system diseases (med=51.4 h) and other diseases (med=51.8 h) compared with those who had neoplasm (med=79.3 h), endocrine, nutritional and metabolic diseases (med=77 h) and digestive system diseases (med=120.1 h) (p<0.05).

Also, LOS had positive significant relationship with age (r=0.222), distance from living area (r=0.081), the number of previous hospitalizations (r=0.058), the number of laboratory tests (r=0.564), radiographies and sonographies (r=0.363) (p≤0.001). On the other hand, LOS had negative significant relationship with the educational level of the patients (r= -0.149) and their husbands (r= -0.104); and with the percent of insurance contribution to total hospital costs (r= -0.061) (p<0.05).

Because of possible interactions among studied factors, they were analyzed simultaneously using Multiple Linear Regression. All qualitative factors including marital status, distance from living area, medical insurance coverage types, admission and discharge months, days and times, final diagnoses, admission types, kinds of the inpatient units, patients educational levels, the spouse jobs and educational levels, related physician specialty and academic degree and patients' status at discharge time (using dummy variables); and quantitative factors including age, the number of diagnostic tests, the number of previous hospitalization and the percent of insurance contribution to total hospital costs which had a relationship with LOS with a significant level less than 0.2 in the univariate analyses were put in the multiple regression model. Then, using Backward method, the associated factors with LOS were determined as following: spouse jobs, admission days, related physicians' academic degrees, final diagnoses and the number of laboratory tests, radiographies and sonographies (p<0.05) (Table 3).

**Table 3:** Associated factors with LOS using Multiple Linear Regression

**Factors**	**B**	**Standardized Coefficients (Beta)**	**P value**
Spouse Jobs			
Worker, farmer, stockbreeder or retired	21.214	0.073	0.001
Admission Days			
Admission on Thursdays	19.547	0.056	0.01
Related Physicians' Academic Degrees			
Being Resident	-18.256	-0.081	<0.001
The Number of Laboratory Tests	13.194	0.435	<0.001
The Number of Radiographies and Sonographies	21.366	0.456	<0.001
Final Diagnoses Category			
Neoplasm	20.504	0.063	0.005

## Discussion

LOS is an important indicator which is used in hospitals extensively. This indicator presents hospital performance and its efficiency level. Therefore, it is an essential indicator to analyze hospital performance. In this survey, we studied the LOS and its association with clinical and non-clinical factors in Women Hospital in Tehran. The results of the simultaneous analysis of the associated factors using Multiple Linear Regression showed that the LOS of patients whose spouses were worker, farmer, stockbreeder or retired was longer than the others' LOS. It can be said that they usually have low socio-economic status and have not appropriate nutrition and health; and cannot use expensive recreation facilities in their lives and may confront more social problems and tensions. Therefore, they seek health care late when their diseases have been extended and need more complex cures. LOS of the patients admitted on Thursdays were longer than that of the others probably because in the next day (weekend in Iran), less diagnostic and curative procedures have been taken and they usually are postponed until the first day of the next week. Therefore, this patient admission process should be changed in order to utilize available hospital resources properly. Cannoodt's and McMullan et al. conducted their studies in the US and Northern Ireland, respectively. They concluded that patients admitted on Fridays and Saturdays have longer LOS. Thus, considering that Fridays and Saturdays are the last days of the weeks in their countries, their results confirm our findings too.[4][15]

Patients who were admitted by residents had shorter LOS compared with those who were admitted by the attending physicians. It was due to either the patients who were admitted by the attending physicians had more serious diseases, or they underwent educational purposes. Moloney et al. reported that the LOS of patients who were admitted by GPs was shorter than that of patients who were admitted by the specialists. However, they did not compare patients' LOS between patients admitted by residents and those admitted by the attending physicians.[24]

Patients who were admitted due to neoplastic diseases had longer LOS that the others; probably because their diseases were more serious and needed more diagnostic and curative procedures. Aguirre-Gas et al. showed that malignant tumors increased patients' LOS.[20] Also, Salesi in his study showed that patients suffered from neoplastic diseases had more LOS than patients suffered from nervous system and digestive system diseases, injury and poisoning.[21] The results of these two studies are consistent with our results. Finally, the results of our study showed that the more the number of laboratory tests, radiographies and sonographies performed for patients, the longer their LOS. It was probably due to the large number of primary routine tests and examinations which have been done in order to diagnose the patients' diseases and the delay occurred in delivering the results from laboratory and radiography units to inpatients units in order to start and continue curative procedures.

As it is shown in Table 3, according to standardized coefficients (Beta), the number of laboratory tests, radiographies and sonographies had more effect on the LOS than the others. The main reasons of the difference between the results of the current study and other mentioned studies are: 1) This survey has been conducted in a women's hospital while other studies have usually been conducted in general hospitals where men and women patients suffered from various diseases were hospitalized; 2) The number of samples in the mentioned studies, compared with our study, has often been small and most of them have suggested that their studies should be conducted with more samples to achieve more accurate results.

The current study had two limitations. This study has been conducted only in a women's hospital and the results probably cannot be generalized to other hospitals, especially general hospitals. Moreover, for collecting data which were not recorded in the patients' charts (such as patients' and their spouse jobs and educational levels, family income), we conducted 951 interviews with all patients admitted in the units in the last three months of the year, because this survey began from the last days of autumn, and related analyses had been carried out only for these patients.

In conclusion, managers and policy makers can only modify some of these associated factors in order to improve patients' LOS. For example, they should prevent Thursdays (weekend in Iran) admissions unless emergency admissions. Also, required primary routine tests and examinations can be performed before admitting patients into the hospital. Further researches are needed to find other factors associated with LOS.

Finally, we suggest conducting studies to find other factors associated with LOS, to show which laboratory tests, radiographies and sonographies are essential and should be performed as routine ones to better determine the patients' health status, and to indicate appropriate rate of LOS in various diseases in Iran to recognize unnecessary and inappropriate hospitalizations and patients' length of stays.
